# Complex opioid driven modulation of glutamatergic and cholinergic neurotransmission in a GABAergic brain nucleus associated with emotion, reward and addiction

**DOI:** 10.1101/2024.12.10.627344

**Published:** 2024-12-11

**Authors:** R. Chittajallu, A. Vlachos, X.Q. Yuan, S. Hunt, D. Abebe, E. London, KA. Pelkey, C.J. McBain

## Abstract

The medial habenula (mHb)/interpeduncular nucleus (IPN) circuitry is resident to divergent molecular, neurochemical and cellular components which, in concert, perform computations to drive emotion, reward and addiction behaviors. Although housing one of the most prominent mu opioid receptor (mOR) expression levels in the brain, remarkably little is known as to how they impact mHb/IPN circuit function at the granular level. In this study, our systematic functional and pharmacogenetic analyses demonstrate that mOR activation attenuates glutamatergic signaling whilst producing an opposing potentiation of glutamatergic/cholinergic co-transmission mediated by mHb substance P and cholinergic neurons, respectively. Intriguingly, this latter non-canonical augmentation is developmentally regulated only emerging during later postnatal stages. Further, specific potassium channels act as a molecular brake on nicotinic receptor signaling in the IPN with the opioid mediated potentiation of this arm of neurotransmission being operational only following attenuation of Kv1 function. Thus, mORs play a remarkably complex role in modulating the salience of distinct afferent inputs and transmitter modalities that ultimately influences synaptic recruitment of common downstream GABAergic IPN neurons. Together, these observations provide a framework for future investigations aimed at identifying the neural underpinnings of maladaptive behaviors that can emerge when endogenous or exogenous opioids, including potent synthetic analogs such as fentanyl, modulate or hijack this circuitry during the vulnerable stages of adolescence and in adulthood.

## Full Text Availability

The license terms selected by the author(s) for this preprint version do not permit archiving in PMC. The full text is available from the preprint server.

