# Supplementary material for: Complex opioid driven modulation of glutamatergic and cholinergic neurotransmission in a GABAergic brain nucleus associated with emotion, reward and addiction
Source: bioRxiv. 2025 Aug 15:2024.12.10.627344. Originally published 2024 Dec 11. Preprint. [Version 2] doi: 10.1101/2024.12.10.627344 (PMC12273893; doi:10.1101/2024.12.10.627344)
Supplement: Supplementary file 1 [file NIHPP2024.12.10.627344v2-supplement-1.pdf]

bioRxiv preprint doi: https://doi.org/10.1101/2024.12.10.627344; this version posted August 15, 2025. The copyright holder for this preprint (which was not certified by peer review) is the author/funder. This article is a US Government work. It is not subject to copyright under 17 USC 105 and is also made available for use under a CC0 license.

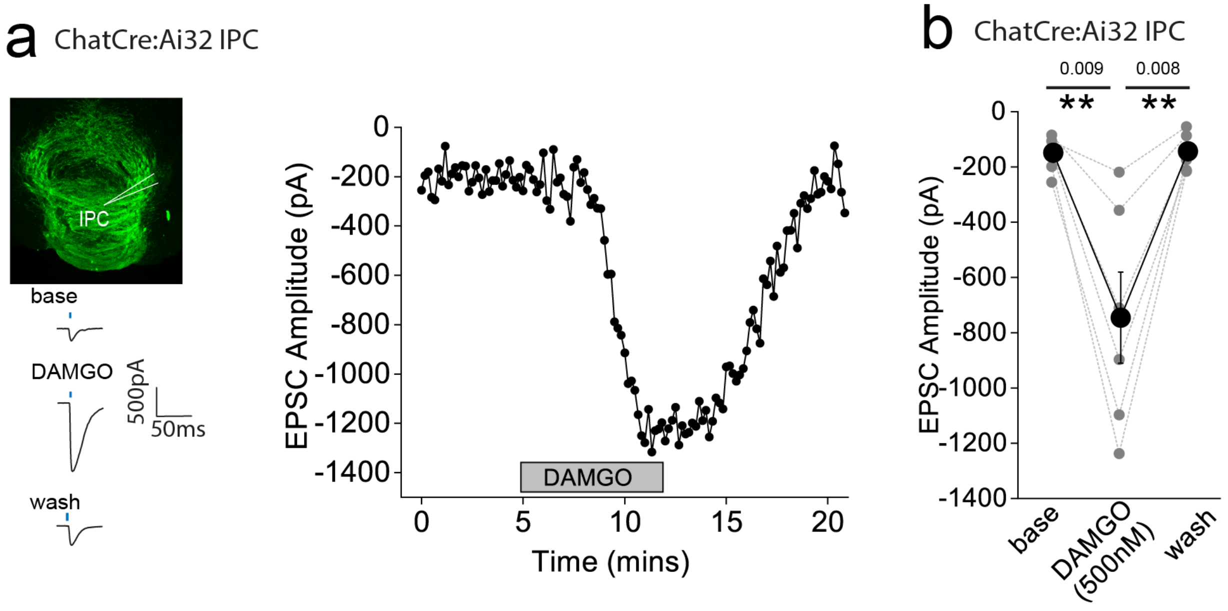

Supplementary Figure 1. AMPAR-mediated synaptic transmission in central IPN (IPC) mediated by mHB cholinergic neurons is potentiated by mOR activation (a) Whole-cell voltage-clamp in adult (>p40) ChATCre:Ai32 mice (top left panel illustrating the axonal arborization of ChR2 expressing cholinergic neuronal axons in IPN and the position of neuronal recording in IPC). Single voltage-clamp traces of light evoked AMPAR EPSCs (bottom left panel; 470nM light pulse; single stimulations, blue dash) and time course of peak amplitudes (right panel) under baseline, during application of 500 nM DAMGO and washout conditions. **(b)** Individual (grey filled symbols) and mean (black filled symbols) data of AMPAR EPSC amplitude (n=8 recorded IPC neurons from 7 mice).

bioRxiv preprint doi: https://doi.org/10.1101/2024.12.10.627344; this version posted August 15, 2025. The copyright holder for this preprint (which was not certified by peer review) is the author/funder. This article is a US Government work. It is not subject to copyright under 17 USC 105 and is also made available for use under a CC0 license.

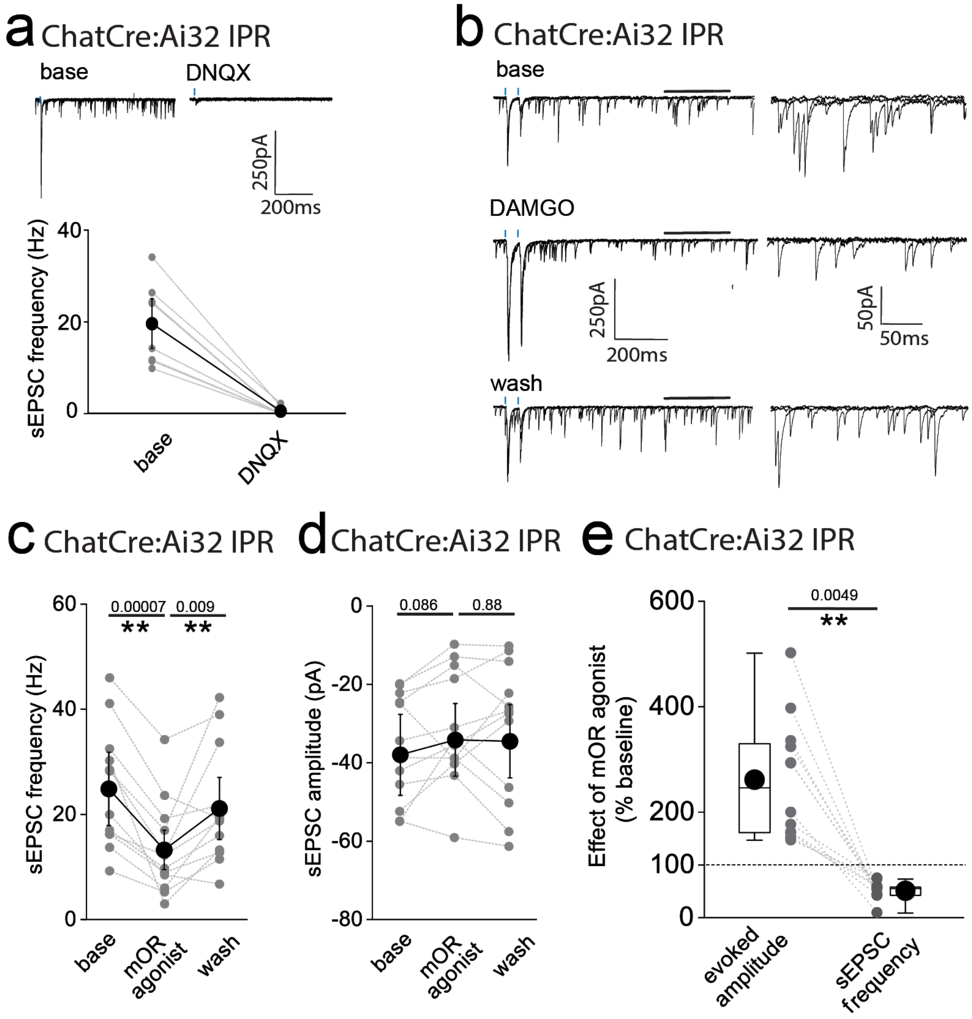

Supplementary Figure 2. mOR activation imparts opposing effects on spontaneous AMPA EPSCs versus cholinergic neuron mediated evoked AMPAR EPSCs in IPR. (a) Single voltage-clamp traces (top panels) illustrating light-evoked AMPAR EPSCs mediated by cholinergic neurons and spontaneous EPSCs (sEPSCs) under baseline and following 10μM DNQX application in an IPR neuron. Individual (grey symbols) and pooled data (black symbols) illustrating complete cessation of sEPSCs as assessed by frequency following 10μM DNQX application (n = 8 recorded IPR neurons from 8 mice; bottom panel). **(b)** Single voltage-clamp traces under baseline, 500nM DAMGO and washout conditions of light-evoked AMPAR EPSCs mediated by mHb cholinergic neurons (2 stimuli at 20Hz; blue dashes) and sEPSCs (left panels). Magnification of sEPSCs events (right panels) corresponding to the region of the traces in the left panels delineated by the black bars. **(c,d)** Individual (grey symbols) and mean (black symbols) data of sEPSC frequency and amplitude (n = 12 recorded IPR cells from 11 mice). **(e)** Box plot and individual data depicting the relative DAMGO mediated percentage change from baseline of the light-evoked AMPAR EPSC peak amplitude versus AMPAR sEPSC frequency in each individual IPR neuron recorded (n = 12 recorded IPR cells from 11 mice).

bioRxiv preprint doi: https://doi.org/10.1101/2024.12.10.627344; this version posted August 15, 2025. The copyright holder for this preprint (which was not certified by peer review) is the author/funder. This article is a US Government work. It is not subject to copyright under 17 USC 105 and is also made available for use under a CC0 license.

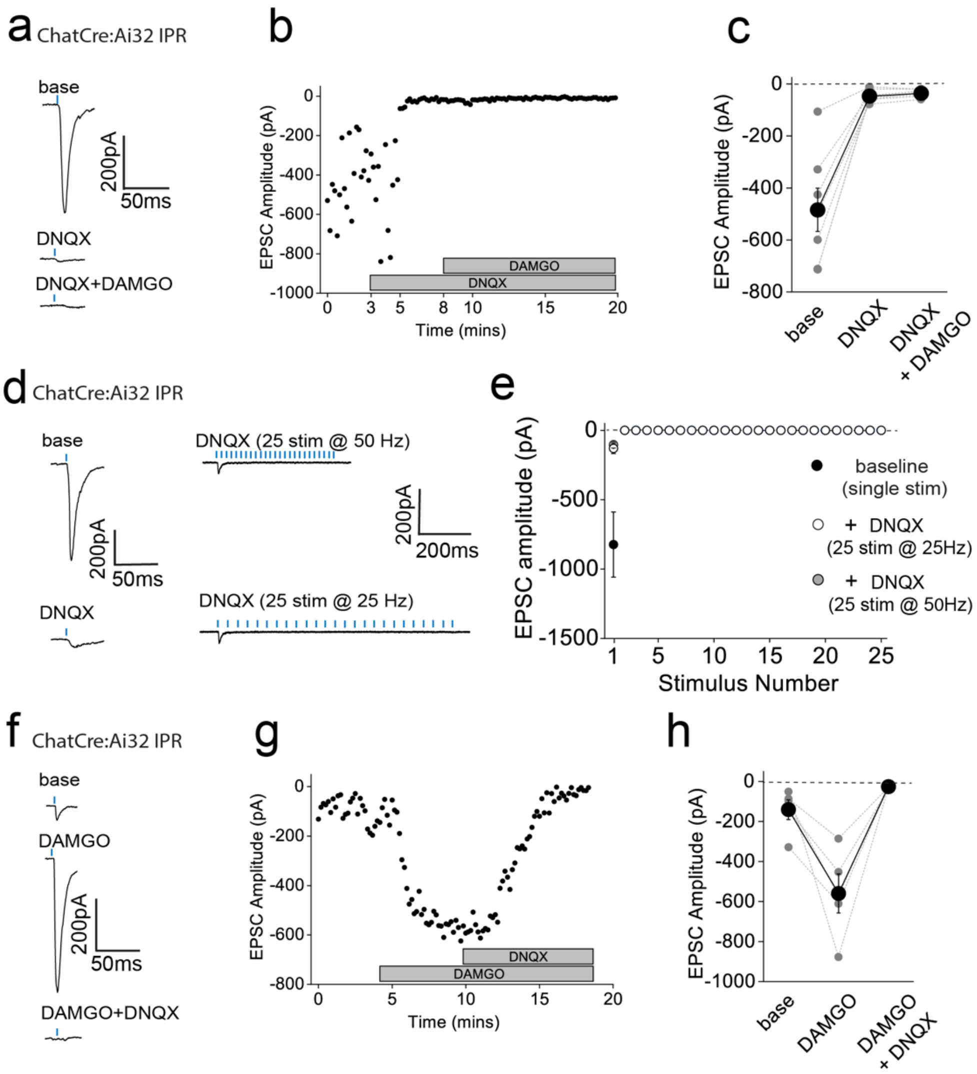

Supplementary Figure 3. Effect on DNQX on synaptic transmission mediated by mHB cholinergic neurons onto IPN prior to or after mOR activation and during high frequency stimulation. (a,b) Voltage-clamp example traces of light-evoked AMPAR EPSCs mediated by cholinergic mHb neurons and time course of peak amplitude under baseline, 10μM DNQX and 10μm DNQX plus 500nM DAMGO conditions. **(c)** Individual (grey filled symbols) and mean (black filled symbols) data of AMPAR EPSC amplitude under baseline, 10μM DNQX and 10μm DNQX plus 500nM DAMGO conditions (n = 7 recorded IPR neurons from 5 mice). (**d**) Voltage-clamp example traces single stimulus light-evoked AMPAR EPSCs mediated by cholinergic mHb neurons stimulation in the presence and absence of 10μM DNQX (left panels). Traces from the same cell in response to trains of light (1ms, 25 stimulations given at 50 or 25Hz; right panels. (**e**) Pooled data of light evoked responses in response to single stimulation (black) and during 50 and 25 Hz trains (grey and open symbols, respectively; n = 5 recorded cells from 2 mice). **(f,g)** Voltage-clamp example trace of single light-evoked AMPAR mediated EPSCs mediated by cholinergic mHb neurons and time course of peak amplitude under baseline, 500nM DAMGO and 500nM DAMGO plus 10μm DNQX conditions. **(d)** Individual (grey filled symbols) and mean (black filled symbols) data of AMPAR EPSC amplitude under, baseline, 500nM DAMGO and 500nM DAMGO plus 10μm DNQX conditions. (n = 5 recorded IPR neurons from 5 mice).
